# Obstetric outcomes in pregnant women with and without depression: population-based comparison

**DOI:** 10.1038/s41598-017-14266-3

**Published:** 2017-10-24

**Authors:** Hui-Chun Huang, Fung-Chang Sung, Pei-Chun Chen, Cherry Yin-Yi Chang, Chih-Hsin Muo, Huei-Sheng Shiue, Jian-Pei Huang, Tsai-Chung Li, Ya-Ling Tzeng, Shu-I Wu

**Affiliations:** 1 Department of Medical Research, Mackay Memorial Hospital, New Taipei, 251 Taiwan; 2 Department of Public Health, China Medical University, Taichung, 404 Taiwan; 3 Center for General Education, MacKay Junior College of Medicine, Nursing and Management, Taipei, 112 Taiwan; 4 Management Office for Health Data, China Medical University Hospital, Taichung, 404 Taiwan; 5 Department of Health Services Administration, China Medical University, Taichung, 404 Taiwan; 6 Department of Obstetrics and Gynecology, China Medical University Hospital, Taichung, 404 Taiwan; 7 Division of Physical Medicine and Rehabilitation, E-Da Hospital, Kaohsiung, 824 Taiwan; 8 School of Medicine for International Students, I-Shou University, Kaohsiung, 824 Taiwan; 9 Department of Medicine, Mackay Medical College, Taipei, 252 Taiwan; 10 Department of Obstetrics and Gynecology, Mackay Memorial Hospital, Taipei, 104 Taiwan; 11 School of Nursing, China Medical University, Taichung, 404 Taiwan; 12 Department of Psychiatry, Mackay Memorial Hospital, Taipei, 104 Taiwan

## Abstract

This study used insurance claims data to evaluate obstetric outcomes in pregnant women with and without depression because population study for Asian women on the issue is limited. We identified 5,064 women with depression at pregnancy in 2005–2013, and 20,024 pregnant women without depression, frequency matched by age, pregnant year and parity. Obstetric events during pregnancy and deliveries were evaluated. The depression group had more events than comparisons for hyperemesis (39.3 vs. 35.5%), abortion (3.3 vs. 2.6%), malpresentation (12.3 vs. 10.3%), C-section (40.2 vs. 34.6%) and intrauterine fetal demise (0.7 vs. 0.4%); risks of these events were significant for childbearing depressed women, not for the 35+ years subgroup. These incidences were higher in depressed women taking antidepressant than those without the medication, but were significant in childbearing depressed subgroup for hyperemesis and C-section with odds ratios of 1.18 (95% confidence intervals (CI), 1.02–1.36) and 1.29 (95% CI, 1.11–1.49), respectively. Incident preterm and low birth weight births were also higher in the depression group than in comparisons, but weren’t significant. In conclusion, women with depression during pregnancy may develop more adverse events than comparisons and are more likely to have a C-section delivery.

## Introduction

The prevalence of depression has increased in recent years, up to approximately 20% of pregnant women reported having depressive disorders^1–3^. Depressive disorders during pregnancy have been associated with negative pregnant outcomes, such as preeclampsia, preterm delivery, premature rupture of membranes, and low infant birth weight. These events lead to neonatal and maternal morbidity and higher healthcare costs^4–9^. There are more studies on neonatal outcomes than studies on other obstetric outcomes associated with maternal depression during pregnancy^10–15^.

Studies on whether depression during pregnancy is associated with increased adverse events on the mother and fetus remain inconclusive. Approximately 15% of women are classified as depressed in a cohort study of 228,876 singleton pregnancies in the Tennessee Medicaid population^2^. The risk of preterm birth and infant convulsions are more common in depressed women who have received medications^12^. A study in Sweden found that planned Cesarean deliveries are more common in women with depression and/or anxiety during pregnancy^5^. A study conducted in Taiwan found that women with depression are less likely to have prenatal care but incur higher expenses for other types of healthcare^16^. Another follow-up study based on a small sample of depressed pregnant women failed to observe significant unfavorable obstetric outcomes^17^. Women may discontinue antidepressant use prior to conception or during the first trimester of pregnancy to ensure safe fetal development^18^. The study of the California County Mental Health System reported that the risk of obstetric complications is lower for depressed women treated in the system than those treated in other settings, with adjusted odds ratios (aORs) of 1.32 and 1.72, respectively, compared with general women^15^. The study of the California County Mental Health System reported that mental illness was associated with elevated risk of obstetric complications; however, the analysis included several types of mental illnesses, and data on depression was not reported separately.

Findings on adverse events for pregnant women with depression are inconsistent. The inconsistency can be related to the limited sample size of studies or the quality of data^19,20^. A recent systemic study analyzed 23 studies covering 25 663 women with untreated antenatal depression reported that these women were more likely to have low birth weight infant and preterm birth^21^. Among these 23 studies, a Korean study failed to find an association between pregnant depression and low birth weight^22^. Information from a population-based study with larger samples for Asian women is limited. A recent study conducted in China reported that prenatal depression combined with anxiety is at a higher risk of having a low birth weight baby^23^. Because of the scarcity of research for Asian population, it remains both opportunity and challenges. In the present study, we used large insurance claims data to investigate whether pregnant women with depression is associated with adverse obstetric outcomes and neonatal outcomes during pregnancy and delivery. The obstetric outcomes were compared between two groups; one group consisted of women with depression and the other group had no such disorder before and during pregnancy. We further assessed whether the association between depression and obstetric complications differs by maternal age at birth and antidepressant medication.

## Results

### Baseline characteristics of study cohorts

Table 1 shows that most women in this study were mainly low-income population in the age of 25–34, living in the northern Taiwan and pregnant for the first time. Women in the depression group were more prevalent with comorbidities than were the non-depression group. Approximately one fifth depressed women were prescribed for antidepressant less than 6 months and only 0.3% depression women were on the medications for longer than 6 months.

**Table 1:** Comparison in demographic characteristics and comorbidities between depressed women and non-depressed women.

	Depressed (N = 5,064)		Non-depressed (N = 20,024)		*p* value
	n	(%)	n	(%)	
Age, years^*^					0.22
<25	578	(11.4)	2311	(11.5)	
25 to 34	3698	(73.0)	14792	(73.9)	
> = 35	788	(15.6)	2921	(14.6)	
Mean (SD)	30.5	(4.7)	30.3	(4.58)	0.02
Monthly Income, NTD					0.07
<15,840	4452	(87.9)	17641	(88.1)	
15,841 to 25,000	445	(8.8)	1620	(8.1)	
>25,000	167	(3.3)	763	(3.8)	
Geographic region					0.0005
North	2069	(40.9)	8726	(43.6)	
Central	1216	(24.0)	4336	(21.7)	
South	1326	(26.2)	5145	(25.7)	
East	453	(9.0)	1817	(9.1)	
Multiple pregnancy					0.07
Yes	65	(1.3)	199	(0.99)	
No	4999	(98.7)	19825	(99.0)	
Previous parity					0.99
0	4372	(86.3)	17268	(86.2)	
1	600	(11.9)	2389	(11.9)	
2	84	(1.7)	336	(1.7)	
≥3	8	(0.2)	31	(0.2)	
Comorbidity					
Diabetes	13	(0.26)	41	(0.20)	0.48
Chronis hypertension	108	(2.1)	279	(1.4)	0.0001
Hyperthyroidism	197	(3.9)	409	(2.0)	<0.0001
Anemia	603	(11.9)	1643	(8.2)	<0.0001
Prenatal care visits					
≤7	676	(13.4)	2661	(13.3)	0.08
8 to 9	1472	(29.1)	5514	(27.5)	
≥10	2916	(57.6)	11849	(59.2)	
Antidepressant medication					
None	4015	(79.3)			
<6 months	1034	(20.4)			
6 months or longer	15	(0.3)			

SD, standard deviation; NTD, new Taiwan dollar, one USD is 30.0–34.0 NTD, recently 30.3 NTD.

### Incidence of obstetric and delivery events

Incident maternal obstetric complications were more likely to occur in the depression group than in the comparison group (Table 2). Significant differences were observed in the multivariable logistic regression analysis. The depressed women were at a greater risk for hyperemesis (adjusted OR = 1.16, 95% CI = 1.08–1.25), abortion (adjusted OR = 1.33, 95% CI = 1.10–1.61), malpresentation (adjusted OR = 1.20, 95% CI = 1.08–1.33), and intrauterine fetal demise (adjusted OR = 1.69, 95% CI = 1.12–2.56). The occurrence of Cesarean sections was 6% higher in the depression group than in the comparison group (40.2% vs. 34.6%, adjusted OR = 1.25, 95% CI = 1.16–1.34). Therefore, women with depression had a lower rate of normal spontaneous delivery (59.2% vs. 65.1%; adjusted OR = 0.80, 95% CI = 0.74–0.85). Compared to the non-depression group, the depression group had higher rates of preterm delivery (6.4 vs. 5.4%) and low birth weight births (2.6 vs. 2.4%), but differences were not significant.

**Table 2:** Odds ratios of maternal obstetric and delivery complications in relation to depression.

	Depressed N = 5064		Non-depressed N = 20024		Odds ratio (95% confidence interval)	
	n	(%)	n	(%)	Crude	Adjusted
Hyperemesis	1988	(39.3)	7117	(35.5)	1.17 (1.10–1.25)***	1.16 (1.08–1.25)***
Gestational diabetes	629	(12.4)	2440	(12.2)	1.02 (0.93–1.12)	1.00 (0.90–1.11)
Preeclampsia/eclampsia	112	(2.2)	439	(2.2)	1.01 (0.82–1.25)	0.92 (0.73–1.17)
Abortion	165	(3.3)	514	(2.6)	1.28 (1.07–1.53)**	1.33 (1.10–1.61)**
Intrauterine growth retardation	19	(0.4)	64	(0.3)	1.18 (0.70–1.96)	1.12 (0.64–1.97)
Antepartum hemorrhage	459	(9.1)	1734	(8.7)	1.05 (0.94–1.17)	1.03 (0.92–1.17)
Placental previa	745	(14.7)	2851	(14.2)	1.04 (0.95–1.13)	0.99 (0.90–1.10)
Placental abruption	54	(1.1)	191	(1.0)	1.12 (0.83–1.52)	1.05 (0.75–1.47)
Premature rupture membranes	395	(7.8)	1491	(7.5)	1.05 (0.94–1.18)	1.08 (0.95–1.22)
Chorioamnionitis	30	(0.6)	99	(0.5)	1.20 (0.80–1.81)	0.94 (0.58–1.54)
Postpartum Hemorrhage	185	(3.7)	688	(3.4)	1.07 (0.90–1.26)	1.11 (0.93–1.32)
Preterm labor /delivery	325	(6.4)	1080	(5.4)	1.20 (1.06–1.37)**	1.12 (0.97–1.29)
Malpresentation	622	(12.3)	2101	(10.5)	1.20 (1.07–1.31)***	1.20 (1.08–1.33)***
Low birth weight	130	(2.6)	488	(2.4)	1.06 (0.87–1.28)	0.97 (0.78–1.21)
Normal spontaneous delivery	3000	(59.2)	13033	(65.1)	0.78 (0.73–0.83)***	0.80 (0.74–0.85)***
Cesarean Section	2036	(40.2)	6924	(34.6)	1.27 (1.19–1.36)***	1.25 (1.16–1.34)***
Vacuum Extraction/Forceps	430	(8.5)	1768	(8.8)	0.96 (0.86–1.07)	0.92 (0.81–1.04)
Intrauterine fetal death	37	(0.7)	87	(0.4)	1.69 (1.15–2.48)**	1.69 (1.12–2.56)*

Adjusted odds ratio was estimated using logistic regression controlling for age, geographic region, chronic hypertension, hyperthyroidism, anemia and medication. *p < 0.05, **p < 0.01, ***p < 0.001.

Table 3 shows the adverse obstetric events were significant mainly for younger depressed groups. The interaction between age and depression was significant only for Cesarean section use (p = 0.03).

**Table 3:** Age-specific odds ratios of maternal obstetric complications in relation to depression.

Age, years	Depressed N = 5064		Non-depressed N = 20024		Odds ratio (95% confidence interval)	
	n	(%)	n	(%)	Crude	Adjusted
<25 yr N	578		2311			
Hyperemesis	265	(45.9)	904	(39.1)	1.31 (1.09–1.57)**	1.29 (1.05–1.59)*
Abortion	18	(3.1)	62	(2.7)	1.17 (0.68–1.99)	1.16 (0.63–2.13)
Malpresentation	53	(9.2)	154	(6.7)	1.41 (1.02–1.96)*	1.48 (1.03–2.13)*
Normal spontaneous delivery	399	(69.0)	1779	(77.0)	0.67 (0.55–0.82)***	0.63 (0.50–0.78)***
Cesarean Section	175	(30.3)	528	(22.9)	1.47 (1.20–1.80)***	1.58 (1.26–1.98)***
Intrauterine fetal death	3	(0.5)	6	(0.3)	2.00 (0.50–8.04)	1.72 (0.34–8.61)
25–34 yr N	3698		14792			
Hyperemesis	1471	(39.8)	5391	(36.5)	1.15 (1.07–1.24)***	1.14 (1.05–1.23)**
Abortion	127	(3.4)	370	(2.5)	1.39 (1.13–1.70)**	1.46 (1.17–1.82)***
Malpresentation	460	(12.4)	1536	(10.4)	1.23 (1.10–1.37)***	1.25 (1.11–1.41)***
Normal spontaneous delivery	2237	(60.5)	9831	(66.5)	0.77 (0.72–0.83)***	0.79 (0.72–0.85)***
Cesarean Section	1448	(39.2)	4914	(33.2)	1.29 (1.20–1.39)***	1.27 (1.17–1.38)***
Intrauterine fetal death	24	(0.7)	61	(0.4)	1.58 (0.98–2.53)	1.63 (0.98–2.71)
> = 35 yr N	788		2921			
Hyperemesis	252	(32.0)	822	(28.1)	1.20 (1.01–1.42)*	1.17 (0.97–1.40)
Abortion	20	(2.5)	82	(2.8)	0.90 (0.55–1.48)	0.93 (0.55–1.56)
Malpresentation	109	(13.8)	411	(14.1)	0.98 (0.78–1.23)	0.99 (0.77–1.26)
Normal spontaneous delivery	364	(46.2)	1423	(48.7)	0.90 (0.77–1.06)	0.92 (0.77–1.09)
Cesarean Section	413	(52.4)	1482	(50.7)	1.07 (0.91–1.25)	1.04 (0.88–1.24)
Intrauterine fetal death	10	(1.3)	20	(0.7)	1.86 (0.87–4.00)	1.88 (0.84–4.20)

Adjusted odds ratio was estimated using logistic regression controlling for geographic region, hypertension, hyperthyroidism, anemia and medication.

Interaction between age and depression was significant for Cesarean section (p = 0.03), but not for hyperemesis (p = 0.44), abortion (p = 0.25), malpresentation (p = 0.12), normal spontaneous delivery (p = 0.06) and intrauterine fetal death (p = 0.90).

### Obstetric events associated with antidepressant use

In the depression group, the use of antidepressant medication was associated with slightly increased events of hyperemesis, chorioamnionitis, preterm birth and Cesarean section use, compared non-users, with an adjusted OR of 2.23 (95% CI = 1.19–4.17) for chorioamnionitis (Table 4). Age-specific analysis shows that the medication relationship was significant in 25–34 years old group for only hyperemesis and Cesarean section use (Table 5). Interaction between age and medication was significant only for Cesarean section (p = 0.04).

**Table 4:** Odds ratios of obstetric and delivery complications in relation to depression with and without antidepressant medication.

	Non-depressed N = 20024		Depression without medication N = 4015		Odds ratio (95% confidence interval)	Depression with medication N = 1049		Odds ratio (95% confidence interval)
	n	(%)	n	(%)	Adjusted	n	(%)	Adjusted
Hyperemesis	7117	(35.5)	1565	(39.0)	1.16 (1.08–1.25)***	422	(40.2)	1.19 (1.05–1.35)**
Gestational diabetes	2440	(12.2)	501	(12.5)	1.00 (0.90–1.11)	128	(12.2)	1.04 (0.86–1.26)
Preeclampsia/eclampsia	439	(2.2)	89	(2.2)	0.93 (0.73–1.17)	23	(2.2)	0.84 (0.54–1.31)
Abortion	514	(2.6)	137	(3.4)	1.33 (1.10–1.61)**	28	(2.7)	1.04 (0.70–1.52)
Intrauterine growth retardation	64	(0.3)	15	(0.4)	1.12 (0.64–1.97)	4	(0.4)	1.11 (0.40–3.05)
Antepartum hemorrhage	1734	(8.7)	364	(9.1)	1.03 (0.92–1.17)	95	(9.1)	1.06 (0.85–1.31)
Placental previa	2851	(14.2)	580	(14.5)	0.99 (0.90–1.10)	165	(15.7)	1.13 (0.95–1.34)
Placental abruption	191	(1.0)	43	(1.07)	1.05 (0.75–1.47)	11	(1.05)	1.03 (0.56–1.88)
Premature rupture membranes	1491	(7.5)	320	(8.0)	1.08 (0.95–1.22)	75	(7.2)	0.98 (0.77–1.25)
Chorioamnionitis	99	(0.5)	19	(0.5)	0.94 (0.58–1.54)	11	(1.1)	2.23 (1.19–4.17)*
Postpartum Hemorrhage	688	(3.4)	155	(3.9)	1.11 (0.93–1.32)	30	(2.9)	0.81 (0.56–1.17)
Preterm labor/delivery	1080	(5.4)	250	(6.2)	1.12 (0.97–1.29)	75	(7.2)	1.33 (1.04–1.70)*
Malpresentation	2101	(10.5)	507	(12.6)	1.20 (1.08–1.33)***	115	(11.0)	1.05 (0.86–1.28)
Low birth weight	488	(2.4)	101	(2.5)	0.97 (0.78–1.21)	29	(2.8)	1.11 (0.76–1.63)
Normal spontaneous delivery	13033	(65.1)	2366	(58.9)	0.80 (0.74–0.85)***	634	(60.4)	0.80 (0.71–0.92)**
Cesarean Section	6924	(34.6)	1625	(40.5)	1.25 (1.16–1.34)***	411	(39.2)	1.24 (1.09–1.42)**
Vacuum Extraction/Forceps	1768	(8.8)	328	(8.2)	0.92 (0.81–1.04)	102	(9.7)	1.10 (0.89–1.36)
Intrauterine fetal death	87	(0.4)	31	(0.8)	1.69 (1.12–2.56)*	6	(0.6)	1.28 (0.56–2.94)

Adjusted odds ratio was estimated using logistic regression controlling for age, geographic region, chronic hypertension, hyperthyroidism, and anemia. *p < 0.05, **p < 0.01, ***p < 0.001.

**Table 5:** Age-specific odds ratios of maternal obstetric and delivery complications in relation to depression with and without antidepressant medication.

Age, years	Non-depressed	Depression without medication		Odds ratio (95% confidence interval)		Depression with medication		Odds ratio (95% confidence interval)
	n	(%)	n	(%)	Adjusted	n	(%)	Adjusted
<25 yr N	2311	438				140		
Hyperemesis	904	(39.1)	201	(45.9)	1.29 (1.05–1.59)**	63	(45.0)	1.26 (0.89–1.77)
Abortion	62	(2.68)	13	(2.97)	1.16 (0.63–2.13)	5	(3.57)	1.34 (0.53–3.40)
Malpresentation	154	(6.66)	42	(9.59)	1.48 (1.03–2.13)*	11	(7.86)	1.18 (0.62–2.23)
Normal spontaneous delivery	1779	(77.0)	295	(67.4)	0.63 (0.50–0.78)***	104	(74.3)	0.86 (0.58–1.28)
Cesarean Section	528	(22.9)	141	(32.2)	1.58 (1.26–1.98)***	34	(24.3)	1.08 (0.73–1.62)
Intrauterine fetal death	6	(0.26)	2	(0.46)	1.72 (0.34–8.61)	1	(0.71)	2.99 (0.35–25.2)
25–34 yr N	14792		2910			788		
Hyperemesis	5391	(36.5)	1152	(39.6)	1.14 (1.05–1.23)**	319	(40.5)	1.18 (1.02–1.36)*
Abortion	370	(2.50)	106	(3.64)	1.46 (1.17–1.82)***	21	(2.66)	1.06 (0.68–1.66)
Malpresentation	1536	(10.4)	372	(12.8)	1.25 (1.11–1.41)***	88	(11.2)	1.07 (0.85–1.34)
Normal spontaneous delivery	9831	(66.5)	1762	(60.6)	0.79 (0.72–0.85)***	475	(60.3)	0.78 (0.67–0.90)**
Cesarean Section	4914	(33.2)	1137	(39.1)	1.27 (1.17–1.38)***	311	(39.5)	1.29 (1.11–1.49)***
Intrauterine fetal death	61	(0.41)	20	(0.69)	1.63 (0.98–2.71)	4	(0.51)	1.17 (0.42–3.24)
> = 35 yr N	2921		667			121		
Hyperemesis	822	(28.1)	212	(31.8)	1.17 (0.97–1.40)	40	(33.1)	1.24 (0.84–1.83)
Abortion	82	(2.81)	18	(2.70)	0.93 (0.55–1.56)	2	(1.65)	0.55 (0.13–2.27)
Malpresentation	411	(14.1)	93	(13.9)	0.99 (0.77–1.26)	16	(13.2)	0.91 (0.53–1.56)
Normal spontaneous delivery	1423	(48.7)	309	(46.3)	0.92 (0.77–1.09)	55	(45.5)	0.91 (0.63–1.31)
Cesarean Section	1482	(50.7)	347	(52.0)	1.04 (0.88–1.24)	66	(54.6)	1.14 (0.79–1.64)
Intrauterine fetal death	20	(0.68)	9	(1.35)	1.88 (0.84–4.20)	1	(0.83)	1.13 (0.15–8.56)

Adjusted odds ratio was estimated using logistic regression controlling for geographic region, chronic hypertension, hyperthyroidism, and anemia. *p < 0.05, **p < 0.01, ***p < 0.001

Interaction between age and medication was significant for Cesarean section (p = 0.04), but not for hyperemesis (p = 0.77), abortion (p = 0.44), malpresentation (p = 0.33), normal spontaneous delivery (p = 0.09) and intrauterine fetal death (p = 0.97).

## Discussion

In the present study, we observed that the baseline comorbidities were more prevalent in the depression group than in the non-depressed group, and the vast majority of depressed women were untreated during pregnancy. This study demonstrates increased events of hyperemesis, abortion, malpresentation, Cesarean Section, and intrauterine fetal death in women with depression during pregnancy. Depressed women aged 25 to 34 had the higher risk of hyperemesis, abortion, malpresentation, Cesarean Section in the age stratified analysis. When examining antidepressant medication, we found depressed women with antidepressant medication did not increase much risk at complications. Hyperemesis and Cesarean Section remained the risk in the childbearing age depressed women with antidepressant medication.

Although this study presented some research feature is differing characteristics among the study groups, as well as other studies, the mechanism of depression associated with adverse obstetric outcomes in pregnant women remains unclear. Women with depression display abnormalities in hypothalamic-pituitary-adrenal axis activity and exhibit high baseline cortisol levels and promoted response to the arginine vasopressin and corticotropin-releasing hormone^24^.

Our data revealed that depressed pregnant women had a higher risk of hyperemesis gravidarum, the risk remained in the childbearing age women with antidepressant medication. However, the risk of hyperemesis gravidarum among childbearing age women was similar in those with antidepressants treatment and those without antidepressants treatment. The reported incidence of hyperemesis gravidarum is relatively rare, varies across ethnic groups, and is dependent on diagnostic criteria^25^. In our study, it is more common because of the diagnostic criteria also included excessive vomiting during pregnancy. A meta-analysis of 59 studies estimated that near 70% of pregnant women experience nausea and/or vomiting and 1.1% with hyperemesis gravidarum^26^. Hyperemesis gravidarum is a primary reason for sick leave and hospitalization during pregnancy^27,28^. Nausea and vomiting of pregnancy (NVP) cause a substantial negative effect on the quality of life for women^29,30^. Wood *et al*. indicated that the effects of NVP are amplified with increased severity of NVP symptoms^30^. Finding in this study echo previous studies investigating the association between depression and hyperemesis^25,31,32^. Bearing in mind that both depression and hyperemesis gravidarum leads to uncomfortable experience among pregnant women. Considerable support should be considered and provided.

Our study also found an increased odds ratio of abortion among the depressed women. The abortion risk may be explained by the decreased levels of Nerve Growth Factor (NGF). Recent studies found that NGF levels are significantly lower among depressed women^33^ and lower NGF level is associated with abortion^34^. NGF is involved in pregnancy maintenance^35^, with an aberrant distribution in placental tissue relevant to pregnancy failure^36^. There have been several studies focused on the association between antidepressant and abortion, yet the study findings are controversial. Most of studies indicated that antidepressants associated with increased risk of abortion^37–39^. A recent review found that there was no statistically significant impact of antidepressant exposure on rates of spontaneous abortion^14^. Similarly, our study observed no significantly increased risk among depressed women without antidepressant. These controversial finding may reflect residual confounding by depression severity and adverse lifestyle^38,40,41^.

The present study also found that depressed women had a higher odds ratio of malpresentation, particularly in those aged 25 to 34. Malpresentation is an important cause of dystocia leading to a risky delivery, and especially for primiparous women^42^. Research on malpresentation is scarce, the etiology is still unclear. Known possible risks include a large baby, polyhydramnios, multiple pregnancy, low-lying placenta, preterm labor, and anomalies of the fetus, uterus or pelvis^43^. When malpresentations occur, primiparous women are at a greater risk for operative delivery than are multiparous women^42^. However, we cannot find out the cause of malpresentation among depressed women in our study, it remains unanswered for the future study.

Consistent with previous studies, pregnant women with depression in our study were at a higher risk of Cesarean delivery^5,6,17,44–47^. The significant risk still remained in the childbearing age depressed women with antidepressant medication in the age stratified analysis. The operation is performed generally for reducing the pain threshold, increasing pain perception, impairing uterine contractility and psychological distress^44^. While experiencing pain, pregnant women with depression had difficulty to function and cooperate properly during labor. Obstetricians may intentionally or unintentionally prefer other operative deliveries due to symptoms of depression^44^. In addition, women might feel anxiety and fear of childbirth and request Cesarean delivery^5^.

In this study, around 80% of depression women are unmedicated during the pregnancy, one fifth depressed women took antidepressants for a short period. This observation is consistent with a large cohort study which shows that most women discontinued antidepressants before the second trimester of pregnancy^12^. Patients who discontinue medications may experience more serious depression symptoms before conception or recurrent depression during pregnancy^48,49^. The potential risk to the infant of antidepressant use during pregnancy is controversial^14^. In our study, the risk of medicated group resembled with un-medicated group. A careful treatment plan is essential for women under antidepressant treatment who plan to conceive or become pregnant.

The present study is strengthened by using a large population data, allowing a high statistical power to capture multiple pregnant outcomes to be compared between depressed women and non-depressed women. We were also capable to stratify data to observe the variation of outcomes by age and antidepressant. This circumstance allowed us not only to investigate the role of depression itself, but also consider the effect of antidepressant medication. In addition, our study focused on pregnancy related outcomes within the pregnancy pathway, rather than delivery or neonatal outcomes. It provided complete information for depressed women and health care professionals to weigh benefits and risks.

This study also has some limitations. Demographic data such as education level, lifestyle history, and marital status, are not available in the data analysis. However, the study sample is unlikely to be affected by these potential confounders because Chinese pregnant women not only avoid unhealthy behaviors but also withdraw the treatment that might harm the fetus. Although our study did not provide the education level of the study sample, levels of income is associated with education. Our data was unable to show a significant difference in income between our study groups and the potential effect is reduced. The second limitation is that information on mental disorders before 1996 was not available, but we were able to ascertain caseness and outcomes based on medical contacts during the follow-up window. However, such measurement errors in both exposure and outcome ascertainments are obscured instead of exaggerated the association of interest. The third limitation is that among depression group only 15 (0.3%) women had the medications for longer than 6 months. We were unable to observe whether women with antidepressant are at a higher risk for complications because of the small sample size. To bridge this gap with adverse outcome, future studies will need to provide enough subjects to evaluate outcomes related to medication.

This population-based retrospective cohort study presented that pregnant women with depression are associated with higher risk of hyperemesis, abortion, malpresentation, and Cesarean Section. These findings have important clinical implications for pregnant women and health care professionals. To make a decision about care during pregnancy, depressed women and clinicians must weigh risks and benefits of treatment options against the consequence associated with untreated illness. The underlying mechanisms on severity of depression, comorbidities, and obstetric outcomes still require further explorations.

## Methods

### Data source and study population

The Department of Health of Taiwan launched a single-payer National Health Insurance (NHI) program in March 1995. Approximately 99% of the 23 million people in Taiwan registered in this program by 2000. With the authorization from the Bureau of National Health Insurance, the National Health Research Institutes of Taiwan established several data files of reimbursement claims publicly available for research.

We obtained from the National Health Research Institutes the Longitudinal Health Insurance Database, containing claims data from 1996 to 2013 for one million persons randomly selected from all insured people. Diagnoses were coded with the *International Classification of Disease*, 9th Revision, Clinical Modification (ICD-9-CM). From the 2005–2013 claims data, we identified pregnant women who had the diagnosis of depression during pregnancy (ICD-9-CM codes of depression: 296.2, 296.20 to 296.25, 296.2, 296.30 to 296.35, 296.82, 300.4, 309.0, 309.1, 309.28, and 311). Only pregnant women with the depression diagnosis for at least 3 times in the outpatient records or for at least once depression diagnosis in the inpatient records were included in the depression cohort. Depressed women with the history of schizophrenia (ICD-9-CM 295), bipolar disorders (ICD-9-CM 296, 296.2, and 296.3) and/or anxiety (ICD-9-CM 300) were excluded. There were 5,064 women eligible for inclusion in the depression group. For each depressed woman, we identified 4 pregnant women without the history of depression, schizophrenia, bipolar disorders and anxiety as the comparison group, frequency matched by age, parity and pregnancy year. Overall, 20,024 women were identified for the non-depressed group. The Research Ethics Committee of China Medical University and Hospital approved this study (CMUH-104-REC2–115). The original identification number of each person in this secondary database has been encrypted and replaced with surrogate identification. The study population was waived from informed consent process because there was no breach of privacy.

### Outcome measures

We examined obstetric adverse outcomes occurred during pregnancy and post-delivery in both groups. We evaluated incident events of hyperemesis, gestational diabetes, preeclampsia/eclampsia, abortion, intrauterine growth retardation, antepartum hemorrhage, placenta previa, placental abruption, premature rupture of membranes, chorioamnionitis, postpartum hemorrhage, preterm delivery, low birth weight, malpresentation, normal spontaneous delivery, Cesarean section, vacuum extraction delivery and intrauterine fetal death.

### Statistical analyses

We used SAS software version 9.4 (SAS Institute Inc., Carey, USA) to perform data analyses for this study. The Chi-square test was used to examine the differences between the depression group and the comparison group for the baseline sociodemographic characteristics, including age (<25, 25–34 and ≥35 years), geographic region of residence (Northern, Central, Southern, and Eastern), monthly income (<15,840 NTD [one USD is equivalent to 30–34 NTD, which is 30.3 NTD recently], 15,841~25,000 NTD and ≥25,001 NTD), pregnancy history, and comorbidities. Comorbidity was considered if the woman was diagnosed at least twice in the outpatient claims or once in the inpatient claims. Diabetes, hypertension, hyperthyroidism, and anemia were the comorbidities included in this study. We measured the number and incidence rate of each of aforementioned pregnancy events for both the depression and comparison groups. Univariate and multiple logistic regression analyses assessed the odds ratio (OR) of each obstetric event associated with the depression status. Both crude OR and adjusted OR with 95% confidence intervals (CI) were presented. Age, geographic region, parity, and comorbidity were included in the multivariable analysis. Age-specific analyses were further performed to identify high-risk age group if the obstetric event appeared significant. Interactions between age and depression were examined for these events. Further data analysis compared the controls against depression with and without medication. Age-specific analyses were also performed to identify high-risk age group if the obstetric event appeared significant. Interactions between age and anti-depressant medication were examined for these events.

### Data availability

The data that support the findings of this study were obtained from National Health Research Institutes (NHRI) of the Ministry of Health and Welfare in Taiwan but restrictions apply to the availability of these data, which were used under license for our study, and so are not publicly available for duplication. Data can be requested only from the Ministry of Health and Welfare.
