# Author Correction: Obstetric outcomes in pregnant women with and without depression: population-based comparison

**DOI:** 10.1038/s41598-018-35248-z

**Published:** 2018-11-08

**Authors:** Hui-Chun Huang, Fung-Chang Sung, Pei-Chun Chen, Cherry Yin-Yi Chang, Chih-Hsin Muo, Huei-Sheng Shiue, Jian-Pei Huang, Tsai-Chung Li, Ya-Ling Tzeng, Shu-I Wu

**Affiliations:** 1 Department of Medical Research, Mackay Memorial Hospital, New Taipei, 251 Taiwan; 2 Department of Public Health, China Medical University, Taichung, 404 Taiwan; 3 Center for General Education, MacKay Junior College of Medicine, Nursing and Management, Taipei, 112 Taiwan; 4 Management Office for Health Data, China Medical University Hospital, Taichung, 404 Taiwan; 5 Department of Health Services Administration, China Medical University, Taichung, 404 Taiwan; 6 Department of Obstetrics and Gynecology, China Medical University Hospital, Taichung, 404 Taiwan; 7 Division of Physical Medicine and Rehabilitation, E-Da Hospital, Kaohsiung, 824 Taiwan; 8 School of Medicine for International Students, I-Shou University, Kaohsiung, 824 Taiwan; 9 Department of Medicine, Mackay Medical College, Taipei, 252 Taiwan; 10 Department of Obstetrics and Gynecology, Mackay Memorial Hospital, Taipei, 104 Taiwan; 11 School of Nursing, China Medical University, Taichung, 404 Taiwan; 12 Department of Psychiatry, Mackay Memorial Hospital, Taipei, 104 Taiwan

Correction to: *Scientific Reports* 10.1038/s41598-017-14266-3, published online 24 October 2017

This Article contains errors in the Methods section under subheading ‘Data source and study population’.

“From the 2005–2013 claims data, we identified pregnant women who had the diagnosis of depression during pregnancy (ICD-9-CM codes of depression: 296.2, 296.20 to 296.25, 296.2, 296.30 to 296.35, 296.82, 300.4, 309.0, 309.1, 309.28, and 311). Only pregnant women with the depression diagnosis for at least 3 times in the outpatient records or for at least once depression diagnosis in the inpatient records were included in the depression cohort. Depressed women with the history of schizophrenia (ICD-9-CM 295), bipolar disorders (ICD-9-CM 296, 296.2, and 296.3) and/or anxiety (ICD-9-CM 300) were excluded.”

should read:

“From the 2005–2013 claims data, we identified pregnant women who had the diagnosis of depression during pregnancy (ICD-9-CM codes of depression: 296.20 to 296.25, 296.30 to 296.35, 296.82, 300.4, 309.0, 309.1, 309.28, and 311). Only pregnant women with the depression diagnosis for at least 3 times in the outpatient records or for at least once depression diagnosis in the inpatient records were included in the depression cohort. Depressed women with the history of schizophrenia (ICD-9-CM 295), bipolar disorders (ICD-9-CM 296, except 296.2, and 296.3) and/or anxiety (ICD-9-CM 300, except 300.4) were excluded.”

